# P-1061. Estimating Transmission and Importation of Healthcare-Associated Pathogens in the Absence of Surveillance Testing: A Bayesian Deep Learning Approach

**DOI:** 10.1093/ofid/ofaf695.1256

**Published:** 2026-01-11

**Authors:** Julia Bohman, Yizhen Xu, Matthew H Samore, Michael Rubin, Karim Khader

**Affiliations:** University of Utah, Salt Lake City, Utah; University of Utah, Salt Lake City, Utah; University of Utah, Salt Lake City, Utah; University of Utah, Salt Lake City, Utah; University of Utah, Salt Lake City, Utah

## Abstract

**Background:**

Accurate estimation of pathogen transmission and importation in healthcare settings typically rely on surveillance cultures, which are costly and not routinely implemented. Clinical cultures, ordered based on symptoms or suspicion are subject to differential test sensitivity, partially capturing asymptomatic colonization, limiting their utility for transmission modeling. We evaluated whether *BayesFlow*, a Bayesian deep learning approach, can estimate key transmission parameters using clinical culture data without active surveillance.

**Figure:**
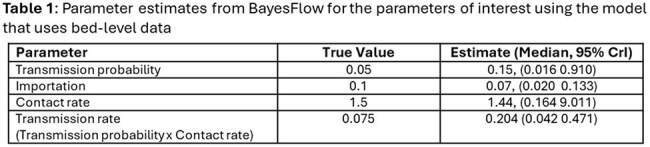

**Figure:**
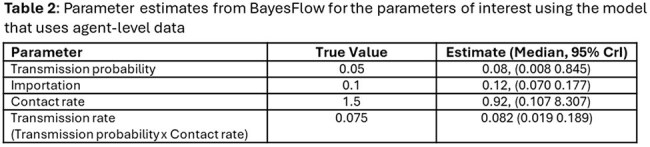

**Methods:**

We simulated pathogen spread in a hospital ward where individuals were either susceptible or colonized. Test rates and culture sensitivity varied by colonization status. We compared two data structures: (1) bed-level tracking of test results per bed over time, and (2) agent-level tracking of individuals' admission and testing. BayesFlow was trained on 10,000 simulations and applied to a dataset with known parameters. We estimated transmission, importation, and contact rate parameters and compared simulated transmission dynamics from true and estimated parameters.

**Figure:**
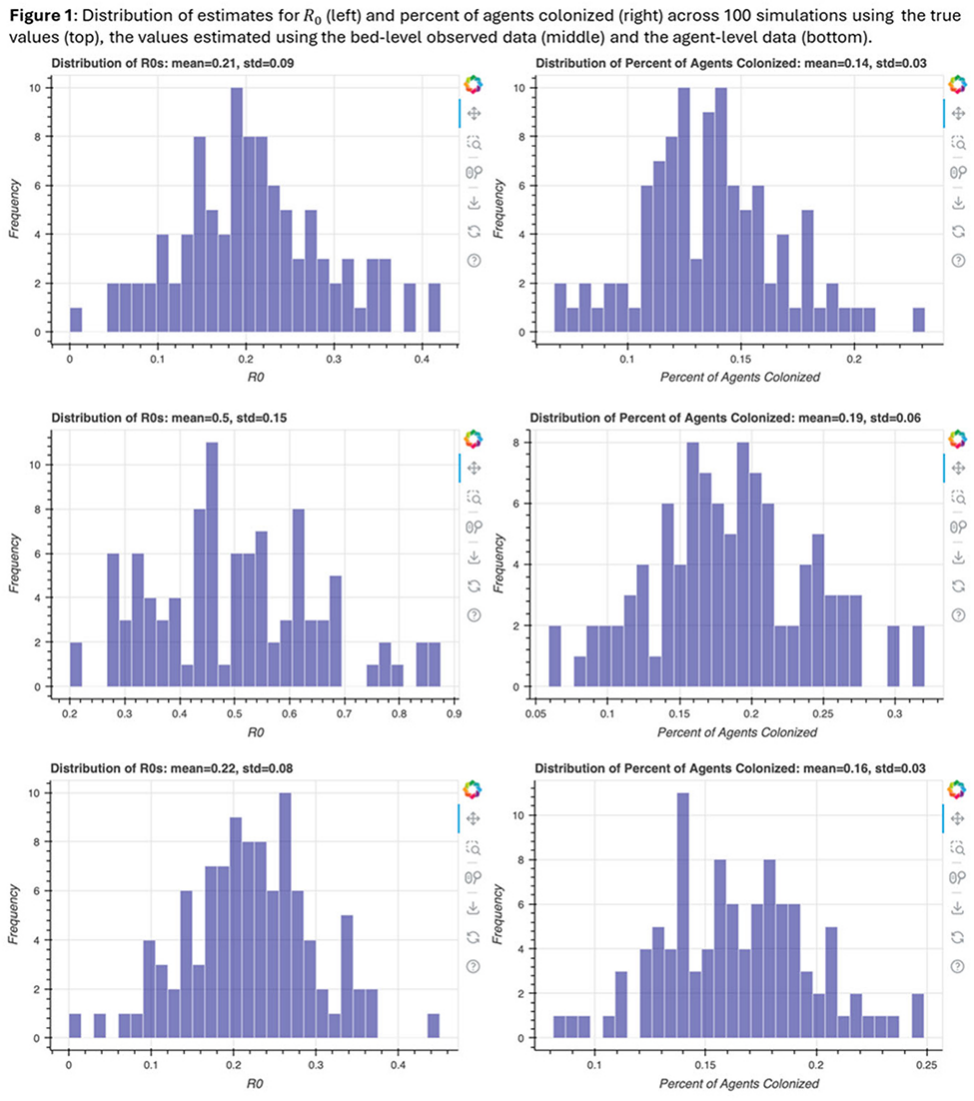

**Results:**

In the Bed-Level structure, BayesFlow substantially overestimated the transmission probability (0.15 vs. true value 0.05) (Table 1), resulting in higher downstream colonizations (Figure 1) while the Fixed Agent structure yielded more accurate estimates across all parameters (Table 2). Simulations based on estimated parameters in this structure produced similar distributions of R₀ and proportion colonized to those generated with true parameters (Figure 1).

**Conclusion:**

BayesFlow shows promise to estimate transmission and importation parameters using only clinical culture data, particularly when using agent-level representations of hospital dynamics, allowing healthcare epidemiologists to infer key drivers of pathogen spread in the absence of surveillance testing. Accurate estimates of transmission dynamics can inform targeted infection prevention interventions, such as contact precautions or cohorting strategies. Next steps include applying this framework to real-world ICU data to assess its utility for monitoring healthcare-associated infection risk and guiding infection control efforts across diverse hospital settings.

**Disclosures:**

Karim Khader, PhD, bioMerieux: Grant/Research Support|Merck: Grant/Research Support

